# Insights into *Penicillium brasilianum* Secondary Metabolism and Its Biotechnological Potential

**DOI:** 10.3390/molecules22060858

**Published:** 2017-06-20

**Authors:** Jaqueline Moraes Bazioli, Luciana Da Silva Amaral, Taícia Pacheco Fill, Edson Rodrigues-Filho

**Affiliations:** 1 Instituto de Química, Universidade Estadual de Campinas, CP 6154, 13083-970 Campinas, Brazil; jaqueline.bazioli@gmail.com; 2 Departamento de Química, Universidade Federal de São Carlos, CP 676, 13.565-905 São Carlos, SP, Brazil; lusamaral@gmail.com

**Keywords:** *Penicillium brasilianum*, secondary metabolism, biotransformation, biological activity

## Abstract

Over the past few years *Penicillium brasilianum* has been isolated from many different environmental sources as soil isolates, plant endophytes and onion pathogen. All investigated strains share a great ability to produce bioactive secondary metabolites. Different authors have investigated this great capability and here we summarize the metabolic potential and the biological activities related to *P. brasilianum*’*s* metabolites with diverse structures. They include secondary metabolites of an alkaloid nature, i.e., 2,5-diketopiperazines, cyclodepsipeptides, meroterpenoids and polyketides. *Penicillium brasilianum* is also described as a great source of enzymes with biotechnological application potential, which is also highlighted in this review. Additionally, this review will focus on several aspects of *Penicillium brasilianum* and interesting genomic insights.

## 1. Introduction

*Penicillium* is a diverse fungal genus with 354 accepted species according to Visagie et al. [1]. This genus is considerable relevant in different scientific fields such as food spoilage, biotechnology, plant pathology and medicine [2], and shows various lifestyles, from necrotrophic pathogenicity to endophytic mutualism. Thousands of *Penicillium* isolates have been screened in bioprospecting programs since the discovery of penicillin, and new bioactive metabolites continue to be discovered from these fungi nowadays.

*Penicillium* genus isolated from studied habitats has been reported to be able to synthesize both previously known and new physiologically active compounds with diverse structures [3]. Some species may produce harmful mycotoxins or cause damages in fruit crops [4], whilst others are considered as great enzyme factories [1]. *Penicillium* species are also capable of producing a diverse assortment of bioactive secondary metabolites, including antibacterial [5,6] and antifungal compounds [7], immunosuppressants and cholesterol-lowering agents [8]. Studies on new bioactive metabolites produced by *Penicillium* species continue to attract attention of researchers nowadays, indicating their current importance as source of novel bioactive molecules to be used by pharmaceutical industry.

In this sense, *Penicillium brasilianum* (synonyms: *Penicillium paraherquei*, *Penicillium ochrochloron* var. *paraherquei*) has been the focus of many researchers in the continuous screening for new bioactive compounds and it has been demonstrated to be an interesting fungus with a great metabolic and underexplored enzymatic potential.

## 2. *Penicillium brasilianum*’*s* Environmental Sources and Isolation Methodologies

Different *Penicillium brasilianum* strains have been isolated from a variety of environmental sources such as plants (endophytes), onion (pathogenic), and soil samples collected from different areas around the world. The next paragraphs present an overview of different sources and places in which *P. brasilianum* has been described.

Geris dos Santos et al. [9] isolated a *Penicillium* sp. strain from the root bark of *Melia azedarach* following the general surface sterilization methodology described by Petrini et al. [10] for endophyte isolation, and cultivated the strain over sterilized rice. Subsequent analysis of internal transcribed spacer (ITS) region of ribosomal DNA confirmed its identity as *P. brasilianum* LaBioMMi 024 [11].

The majority of *P. brasilianum* strains reported in the literature are isolated from soil. Fujita et al. [12] described the metabolism of *P. brasilianum* JV-379 strain that was collected from a soil sample around Sakai (Osaka, Japan). The strain was identified as *Penicillium brasilianum* Batista based on its morphological characteristics. A few years later, Schurmann et al. [13] described the isolation of *Penicillium brasilianum* as a soil fungal species which was collected at the Serra do Cipó National Park, in Minas Gerais State (Brazil).

*Penicillium brasilianum* from Korean soil was also described by Cho et al. [2]. The *Penicillium* species was isolated using the soil dilution plate method, and later identified based on the morphological and molecular phylogenetic analyses [2]. *P. brasilianum*’s sequence analysis of β-tubulin gene presented similarity ranged from 70.0–95.7%.

Researchers from Iraq also identified *P. brasilianum* in soil samples isolated by dilution plate method a few years later. The isolation of different genera of fungi from 80 soils samples in nine stations was made in Salahaddin Province, north of Baghdad, Iraq [14]. According to the preliminary study, *Penicillium brasilianum* Batista isolate obtained from Balad area soil filtrate was the most effective isolate against the bacteria strains tested in the study.

*Penicillium* is one of the most used genera in biotechnology and *Penicillium brasilianum* is also a very important biotechnological target for enzyme production as reviewed here [15,16,17]. Jørgensen et al. [15] described a series of biotechnological enzymes produced by an isolate of *P. brasilianum* IBT 20888 isolated from seaweed in Denmark. Zeni et al. [16] reported pectinase-producing microorganisms with polygalacturonase activity including the isolated *Penicillium* sp. from tea, which was posteriorly identified as *Penicillium brasilianum* by molecular biology techniques [17].

*P. brasilianum* was also reported for the first time as a new fungal pathogen of onion bulbs (*Allium cepa* L.) based on ITS region, β-tubulin region, and elongation factor 1-α gene sequences. The isolated *P. brasilianum* was able to infect both the inner and outer layers of onion bulbs and be re-isolated from the infected tissues. Although many fungal pathogens of onion bulbs have been studied, no occurrence of *P. brasilianum* as a fungal pathogen had been reported for over the past years [18].

## 3. Secondary Metabolites Production in *Penicillium brasilianum*

The genus *Penicillium* is a rich and diverse source of bioactive compounds [18], which vary in structure and are synthesized via different biosynthetic pathways [19]. *P. brasilianum* isolates have been proven this great synthetic ability. In total, 40 secondary metabolites were described to be biosynthesized by this *Penicillium* species (Table 1), including alkaloid-nature metabolites, diketopiperazines, meroterpenoids, polyketides and cyclodepsipeptides. Structurally interesting brasiliamides [11,20], austin-related insecticidal meroterpenes [9,13,21], verruculogen like tremorgenic alkaloids [22] and spirohexalines, which are new inhibitors of bacterial undecaprenyl pyrophosphate synthase have been isolated by this *Penicillium* species so far [23].

Geris dos Santos and Rodrigues-Fo, described the isolation of *P. brasilianum* as endophyte from the root bark of *Melia azedarach* (Meliaceae), and reported the production of a series of meroterpenoids [9]. Meroterpenes are secondary metabolites most often isolated from fungi and marine organisms, frequently associated to *Penicillium* and *Aspergillus* genera, but also produced in higher plants [39]. These compounds are characterized by its mixed biosynthetic origin, which are partially derived from terpenoids. Austin (**1**) is a good representative of meroterpenoid compounds and it was isolated for the first time in 1976 by Chexal et al. [40] from an *Aspergillus ustus* culture, and later found in *Penicillium* sp. MG-11 [24] and in a different strain of *P. brasilianum* along with six other meroterpenes **2**, **3**, **6**, **7**, **8** and **25** (see Table 1) [24]. The Austin-related meroterpenoids described in *P. brasilianum* were associated with different biological activities. Clardy and co-workers studied the convulsive activity of the meroterpenoids dehydroaustin (**6**), austin (**1**), and acetoxydehydroaustin (**7**) against silkworms. The convulsive activity of the meroterpenoids was examined individually in the presence or absence of verruculogen (**25**), which typically caused silkworm convulsions only at a dosage of more than 0.1 µg/g of diet. The result showed that dehydroaustin alone at dosages of 1–100 µg/g of diet did not cause silkworm convulsions. Austin and acetoxydehydroaustin also exhibited no effect. However, when these compounds were applied in combination with verruculogen, they were able to enhance the convulsive effect of verruculogen against the silkworm. Among them, acetoxydehydroaustin showed the highest enhancement [24]. Thus, austin-related compounds were reported for the first time to increase the effect of verruculogen in silkworms. Geris et al. [26] also reported the production, isolation and identification of three new meroterpenes, named preaustinoid A1 **11**, A2 **12** and B1 **15**, produced by *Penicillium brasilianum* LaBioMMi 024 associated with *Melia azedarach*.

Geris dos Santos and Rodrigues -Fo in further chemical studies of *P. brasilianum* LaBioMMi 024 reported two austin-like meroterpenoids, preaustinoid A and B (**10** and **14**, respectively), in addition to the known alkaloid verruculogen (**25),** when the strain was cultivated in rice. The compounds exhibited moderate bacteriostatic effects on *Escherichia coli*, *Staphylococcus aureus*, *Pseudomonas aeruginosa* and *Bacillus* sp. [9]. Preaustinoid A (**10**) was later crystallized and the structure and absolute configuration determined [30]. According to the authors the four fused rings are in different distorted conformations. Rings A and C are distorted towards a half-chair conformation, ring B is distorted towards a half-boat conformation, and ring D is a boat conformation that is highly distorted towards a half-boat. Few years later, Fill et al. [11] reported the isolation and structural characterization of the modified meroterpenoids preaustinoid A1 (**11**), preaustinoid B2 (**16**) and austinolide (**4**) obtained from *P. brasilianum* LaBioMMi 024. Preaustinoid A and A1 were evaluated as inhibitors of the signaling enzyme caspase-1 indicating the potential as inhibitors of interleukin 1-β production by inflammasomes in induced THP-1 cell line assays [41].

The antibacterial activity of the meroterpenes preaustinoid A and B (**10** and **14**, respectively) presented bacteriostatic effect for all microorganisms tested at a dosage of 250 μg/mL. However, the bactericidal effect was observed only with the compound preaustinoid A (**10**) on *E. coli, P. aeruginosa* and *Bacillus* sp. at dosages of 250 μg/mL. In addition, the compounds preaustinoid A **10** and verruculogen (**25**) presented this effect at dosages of 125 and 250 μg/mL respectively to *E. coli* [9].

Schürmann et al. [13] described the meroterpenes austin (**1**) and dehydroaustin (**6**) isolated from *Penicillium brasilianum* Batista found in soil at the Serra do Cipó National Park, in Minas Gerais State (Brazil). The activity of the extract and isolated compounds was tested against six bacteria and for acetylcholinesterase inhibition [13]. However, neither austin nor dehydroaustin presented activity towards any of the tested bacteria. Other significant compounds were isolated from *P. brasilianum* by Schürmann et al. [13], such as penicillic acid (**23**) and the polyalcohol d-mannitol (**24**). The authors reported the antibacterial assay by disc diffusion of penicillic acid (100 μg/mL) as active against the pathogenic bacteria strains *S. aureus* (IZ = 18 mm), *L. monocytogenes* (IZ = 26 mm), *B*. *cereus* (IZ = 15 mm), *S*. *typhimurium* (IZ = 15 mm), *E*. *coli* (IZ = 15 mm) and *C*. *freundii* (IZ = 16 mm). Therefore, the authors reported penicillic acid as active against *S. aureus*, *L. monocytogenes*, *B. cereus*, *S. typhimurium*, *E. coli* and *C. freundii* showing MIC values of 512 μg/mL against *S. aureus* and *S. typhimurium*, and 256 μg/mL against *L. monocytogenes*, *B. cereus*, *S. typhimurium*, *E. coli* and *C. freundii* [13].

Penicillic acid (**23**) is also described as an important phytotoxin produced by *Penicillium cyclopium* and *Penicillium canescens* presenting high herbicide activity manifested by its ability to alter the germination of corn [42]. The experiments indicated that the percentage of inhibition of corn seeds germination was directly proportional to the logarithm of the penicillic acid concentration. Penicillic acid is also described to affect the germination of fungal spores, and affect the overall turnover of the metabolites in *Zea mays* [42,43]. On the other hand, polyalcohols profile has been pointed out as a quimiotaxonomic marker for fungi, and d-mannitol is considered the most abundant of all the soluble polyalcohols found in fungi [25]. It has been reported that d-mannitol inhibits an angiotensin I converting enzyme (ACE). The anti-hypertensive effect of d-mannitol was also demonstrated in spontaneously hypertensive rats (SHR) by oral administration [32].

Kataoka et al. [29] studied the ability of austin (**1**) and its derivatives dehydroaustin (**6**) and acetoxydehydroaustin (**7**) to selective blocking action on cockroach nicotinic acetylcholine receptors, and the ability to paralyze male adult American cockroaches. The paralysis of cockroaches was observed within 1h after injection [29]. The authors concluded that the Austin family compounds act selectively as antagonists on neuronal nicotinic acetylcholine receptors. 

The toxicity of some austin related meroterpenoids towards insects were investigated by Geris et al. [28]. The authors found a direct insecticidal action of dehydroaustin (**6**), and acetoxydehydroaustin (**7**), against the third-instar larvae of dengue fever vector *Aedes aegypti*. They evaluated the tested lethal concentrations (LC_50_ and LC_90_) of the compounds, and 24 h after exposure, observed that dehydroaustin was the most active meroterpenoid in the series, with an LC_50_ value of 2.9 ppm, making it an attractive natural insecticide [28].

In 1983, Okuyama and co-workers, described the isolation and characterization of the compound paraherquonin (**39**) from the cultures of *Penicillium paraherquei* IFO 6234, a synonym of *P. brasilianum.* The new meroterpenoid with a unique hexacyclic skeleton had its chemical structure determined by X-ray diffraction and NMR data analysis [33]. The authors evaluated the intravenous injection of this compound at 100 mg/kg to mice and paraherquonin has shown no lethal effect. Recently, Matsuda and co-workers investigated the biosynthetic mechanisms and the gene cluster responsible for the production of paraherquonin in *P. brasilianum* NBRC 6234 (*prh* cluster). The authors further described and characterized the pathway leading to berkeleydione (**40**), a metalloprotease-3 and caspase-1 inhibitor [34,44], which involves four oxidative enzymes, one of them, a nonheme iron-dependent dioxygenase PrhA responsible for the cycloheptadiene formation to yield berkeleydione [38]. Interestingly, the authors noted that another *P. brasilianum* strain (*P. brasilianum* MG11), which is already described as a producer of austin and acetoxydehydroaustin, [29] has a homologous gene cluster to the *prh* cluster encoding a predicted dioxygenase that is almost identical to PrhA (*aus* cluster). Matsuda and co-workers were able to prove that the homologous dioxygenase encoded by the *aus* cluster shares its substrate with PrhA but produces a different product with a spiro-lactone moiety, diverging the metabolic pathways for the two different compounds in the same species [38].

*Penicillium brasilianum* is described as a great producer of bisphenylpropanoid amides named brasiliamides [20]. Brasiliamides A and B were firstly identified as secondary metabolites from *P. brasilianum* Batista JV-379 isolated from soil [12]. The convulsive activity of brasiliamides A (**17**) and B (**18**) was examined with the third instar larvae of silkworm (*Bombyx mori*), and evaluated as ED_50_ values of 300 and 50 μg/g of diet, respectively, upon oral administration [12]. Further investigations by Fujita and co-workers, resulted in the isolation of three new brasiliamide congeners, named brasiliamides C, D and E (compounds **19**, **20**, **21**, respectively, Table 1) from okara fermented with *Penicillium brasilianum* Batista JV-379 [20]. The isolation and structural elucidation of brasiliamides, their rotational properties in solution, and their convulsive activity against silkworms were also evaluated. Both brasiliamides C and D showed convulsive activity against silkworms with an ED_50_ value of 400 μg/g of diet, whereas brasiliamide E showed less activity than the others. Fill et al. [11] obtained a new, slightly modified congener named brasiliamide F (**22**). The authors submitted the amides for their antimicrobial activity against a set of pathogenic bacteria, however, only brasiliamide A, the major amide obtained, showed weak inhibitory effects to *Bacillus subtilis* with a MIC of 250 µg/mL [11].

Verruculogen (**25**) was reported by Geris dos Santos and Rodrigues-Fo [9] for the first time as a secondary metabolite produced by *P. brasilianum*. The alkaloid is one of the tremorgenic mycotoxins produced by fungi belonging to the genera *Penicillium* and *Aspergillus* that elicit intermittent or sustained tremors (staggers syndromes) in vertebrate animals [45,46]. In recent investigations, Fill et al. [47] reported that verruculogen (**25**) exhibited weak antiparasitary activity against *Leishmania amazonensis*. Further studies by Fill et al. [22] described the production of further indole alkaloids by the same endophytic strain of *P. brasilianum* cultivated in rice. In addition to the production of verruculogen (**25**), the compound TR-2 (**26**) and a verruculogen TR-2 C-11 epimer **27** were isolated and characterized in further studies. Verruculogen TR-2 (**26**) was described for this species for the first time and the C-11 epimer of verruculogen TR-2 (**27**), was described as a novel fungal natural product. [22].

Tang et al. [35] reported six known compounds produced by *P. brasilianum*, isoroquefortine C (first report as a naturally occurring compound), griseofulvin, ergosterol peroxide, β-hydroxy-(22*E*,24*R*)-ergosta-5,8,22-trien-7-one, cerevisterol and (22*E*,24*R*)-6β-methoxyergosta-7,22- diene-3β,5α-diol **28**, **29**, **30**, **31**, **32** and **33**, respectively. The compounds were investigated concerning the bioactivity against five phytopathogenic fungi (*Gibeberalla saubinetti*, *Fusarium solani*, *Botrytis cinerea*, *Colletotrichum gloeosporioides* and *Alternaria solani*) and four pathogenic bacteria (*Escherichia coli*, *Bacillus subtilis*, *Staphyloccocus aureus* and *Bacillus cereus*). The authors also investigated the allelopathic activities on *Raphanus sativus*. Isoroquefortine C exhibited antifungal activity against *C. gloeosporioides*, *A. solani* and *B. cinerea*, with MICs of 12.5, 25 and 50 μM, respectively. Griseofulvin exhibited moderate to strong activity against Gram-positive bacteria *S. aureus*, *B. cereus* and *B. subtilis* (MICs of 3.13–25 μM), and also showed strong inhibitory effects on the growth of *A. solani* and *S. aureus* with MIC of 3.13 μM for each [35]. This was the first report of these metabolites produced by this fungus, and have proved the high biological activities related to *P. brasilianum*’s secondary metabolites production.

Furthermore, Fill et al. [47] in their continuous studies concerning the chemistry and biochemistry of *P. brasilianum*, described that small modifications in the culture medium composition altered the secondary metabolite profile of *P. brasilianum* [47]. The effect of different cultivation conditions by the addition of CaCl_2_, CuSO_4_, glycerol, KCl, MnSO_4_ concerning the metabolic profile for the strain *P. brasilianum* LaBioMMi 136 was evaluated. They observed that medium composition supplemented with CuSO_4_ and MnSO_4_ locked verruculogen biosynthesis and addressed the amino acid proline to the production of a series of cyclodepsipeptides identified as JBIR 113, JBIR 114 and JBIR 115, never described for this species so far, indicating the great enzymatic machinery potential of this fungus species for the production of peptide related metabolites. The peptides were initially described by Kawahara and co-workers, to be produced by a marine sponge-derived *Penicillium sp.* fS36 isolated in Japan [48]. The unique structure with three neighboring cyclic amino acids proline and twice pipecolinic acid is rare as natural products and has been described for the first time in a terrestrial organism. The effect of the cyclodepsipeptide JBIR 113 was evaluated in promastigotes of *L. amazonensis* and epimastigotes of *T. cruz* [47] and was found inactive in the bioassays exhibiting an IC_50_ value (inhibitory concentration for 50% of the parasites) of 63.2 ± 2.5 µM. The compound was also found to be inactive concerning antimicrobial activity against *Pseudomonas aeruginosa*, *Staphylococcus aureus* or *Escherichia coli*. The three cyclodepsipeptides were also investigated concerning the cytotoxicity, however none of them showed cytotoxicity to human cervical carcinoma HeLa cells lines (IC_50_ ≥ 100 mM) [48]. 

Inokoshi et al. [23] described the isolation of spirohexaline (**37**) and viridicatumtoxin (**38**) from the culture broth of *P. brasilianum* FKI-3368. Spirohexaline and viridicatumtoxin inhibited undecaprenyl pyrophosphate (UPP) synthase activity with IC_50_ values of 9.0 and 4.0 μM, respectively [23]. Studies performed by Inokoshi and co-workers indicated that spirohexaline and viridicatumtoxin show antimicrobial activity, due to the inhibition of bacterial undecaprenyl pyrophosphate UPP synthase. They appear to be ideal UPP synthase inhibitors because of the good correlation between the inhibition of UPP synthase and antibacterial activity. Bacterial UPP synthase is recognized as a promising target for the development of new anti-infectious agents that are effective against resistant bacteria due to its essential role in the biosynthesis of peptidoglycan and other cell-wall polysaccharide components. Therefore, spirohexaline is a good candidate for the development of a new type of anti-infectious agents [36]. Inokoshi et al. [37] described that these small fungal molecules **37**, **38** showed potent antibacterial activity, particularly against Gram-positive bacteria including methicillin-resistant *Staphylococcus aureus* (MRSA), fungi and yeasts. However, viridicatumtoxin was generally more potent than spirohexaline. In the same study, the authors performed a molecular docking approach in order to propose a working hypothesis of the inhibitory mechanism between the two inhibitors and bacterial UPP synthase. Furthermore, the molecular modeling suggested that the hydrophobic spirobicyclic ring of viridicatumtoxin interacts with three hydrophobic clefts of the active site in MRSA UPP synthase [37]. Viridicatumtoxin was originally isolated as a mycotoxin [49]. The single-dose LD_50_ in mice was described as 122.4 mg/kg in the initial report, but nonlethal oral administration up to 350 mg/kg in mice was described in a later study [50]. Bladt and co-workers [51] reported a screening of 289 fungal extracts in order to characterize fungal natural products (NPs) with in vitro bioactivity towards chronic lymphocytic leukemia (CLL) cells. One of the studied strains was *P. brasilianum,* and viridicatumtoxin was isolated as one of the most cytotoxic compounds tested towards CLL cells in the screening experiments. The authors described a median lethal concentrations (LC_50_) value between 0.7 and 3.5 nM.

## 4. Biotechnological Potential of *P. brasilianum*

**Figure 1:**
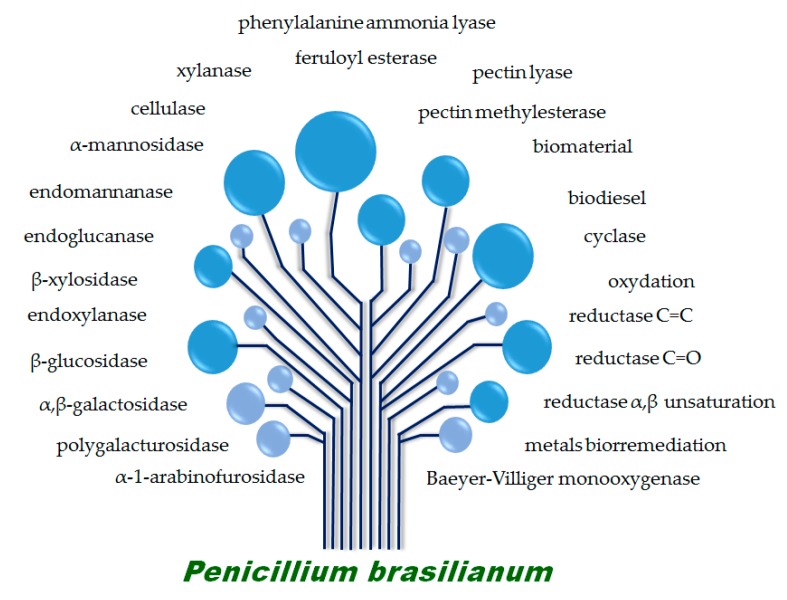
The biotechnological potential of *Penicillium brasilianum.*

The industrial exploration of secondary metabolites requires knowledge concerning the genetic basis of the biosynthesis, biosynthetic enzymes and regulatory genes, in order to develop metabolic engineering strategies for optimizing the production of a natural product of interest and making the process economically feasible [52]. In addition, the biosynthetic enzymes may also be used in important biotechnological processes. Understanding the genetic foundation of secondary metabolite biosynthesis further allows systematic generation of “unnatural” natural products by manipulating the genes involved and redesigning the pathways resulting in structural diversity and potential biological activities. Recently, Nielsen et al. published a great paper concerning a global analysis of biosynthetic gene clusters in *Penicillium* species and interestingly the analysis of 24 *Penicillium* genomes identified an even greater unexploited potential for secondary metabolites production by this genus than expected [53]. Besides the great ability of *P. brasilianum* to produce novel bioactive secondary metabolites, this species has also been described as a great source of enzymes with biotechnological potential (represented by Figure 1) and biotransformation purposes.

Thygesen et al. [15] firstly studied the production of cellulose and hemicellulose-degrading enzymes by *Penicillium brasilianum* IBT 20888 isolated from seaweed in Denmark, which was cultivated on wet-oxidized wheat straw. Cellulases and hemicellulases (glycosylhydrolases) are produced by a range of microorganisms, including bacteria, actinomycetes, and yeasts, however, fungi appear to be the most efficient producers of extracellular enzymes [54,55]. *P. brasilianum* seems to be a very interesting producer of endoglucanase and β-glucosidase, with high enzyme activities of 1.34 FPU/mL. Enzymatic hydrolysis of filter cake from wet-oxidized wheat straw, resulted in glucose yields from cellulose of 58% (*w*/*w*) and 39% (*w*/*w*) using enzymes produced by *P. brasilianum* (5 FPU/g biomass) and a commercial mixture of Celluclast and Novozyme 188 indicating that the enzymes from *P. brasilianum* are as efficient as the commercial enzyme mixture in hydrolyzing this substrate [15]. Interestingly, the authors reported that with a higher enzyme loading (25 FPU/g biomass), the glucose conversion from cellulose was between 77–79% (*w*/*w*) indicating the great biotechnological potential of this *Penicillium* species. 

In further studies concerning the biotechnological potential of *Penicillium brasilianum* IBT 20888, Jørgensen et al. [56] described the cultivation of this strain on a medium containing both cellulose and xylan in order to induce a wide range of enzymes. The authors reported the activities of endoglucanase, endoxylanase, endomannanase, β-glucosidase, β-xylosidase, α-l-arabino-furanosidase, and α-galactosidase as well as further purification of five cellulases and one xylanase, which together constituted around 78% of the total protein of *P. brasilianum* produced in the tested medium. The purified enzymes were studied on different substrates for classification concerning activity. The hydrolysis studies revealed two cellulases acting as cellobiohydrolases (active on microcrystalline cellulose, Avicel), three of the cellulases were active on both Avicel and carboxymethyl cellulose indicating endoglucanase activity. In addition to endoglucanase activity, two of these showed also mannanase activity. The authors also reported the purification of a basic xylanase (pI > 9) active towards xylan. 

In 2004, Jørgensen et al. [57] published an interesting investigation concerning the effect of various monosaccharides as carbon source on the growth and production of cellulases and xylanases produced by *P. brasilianum* IBT 20888. The fungus was studied on a mixture containing glucose (10 g/L), arabinose (5 g/L) and xylose (5 g/L). The results indicated that glucose was the first sugar to be metabolized by the strain and no uptake of the other sugars was observed before glucose depletion [57]. After that, mannose, xylose and galactose started to be metabolized. Glucose was also found to repress the production of cellulases and xylanases and during exponential growth on glucose no detection of endoglucanase, β-glucosidase, endoxylanase or β-xylosidase activity was observed. Xylose did not repress the enzyme production, however, it induced the production of endoxylanases and β-xylosidases.

Furthermore, Jørgensen and Olsson reported in 2006 [58] the effect of substrate utilized in the cultivation of *Penicillium brasilianum* IBT 20888 and the hydrolytic performance of the enzyme preparations. Later, the same research group investigated the production of arabinoxylan-degrading enzymes by the same *P. brasilianum* strain. In the study, they observed, under solid-state fermentation (SSF) and optimum growth conditions, the maximum production of feruloyl esterase, xylanase, and α-l-arabinofuranosidase. After optimizing the conditions concerning initial pH, temperature, and nitrogen source content (80% moisture, pH 6, 26.5 °C, and 5 g L^−1^ nitrogen source), the authors reported the maximum level of feruloyl esterase of 1542 mU/g BSG that was observed after 196 h, while xylanase (709 U/g BSG) and ArabF activity (3567 mU/g BSG) indicated a maximal after 108 h and 96 h, respectively [59]. Feruloyl esterases (FAEs) represent a diverse group of carboxyl esterases that specifically catalyze the hydrolysis of ester bonds between ferulic (hydroxycinnamic) acid and plant cell wall polysaccharides [60]. Thus, the production of sidegroup cleaving enzyme activity like FAEs is very important for the complete enzymatic hydrolysis of agro-industrial by products [61]. The specificity profile of *P. brasilianum* crude extract against the hydroxycinnamic acid methyl esters suggests that it contains only type-B feruloyl esterases [59]. The authors identify *Penicillium brasilianum* as a promising fungus for the production of both sidegroup cleaving and depolymerizing enzyme activities for the complete degradation of arabinoxylan [59].

Panagiotou et al. [62] described *P. brasilianum* as an enzyme factory, highlighting the essential role that FAEs play in the hydrolysis of the plant cell wall. The authors studied and reported the influence of carbon source, initial pH (pH 4–9), nitrogen source, and cultivation temperature (24–36 °C) on the production of the enzymes FAE, xylanase and α-l-arabinofuranosidase by *P. brasilianum*. From the results obtained, the authors reported a great feruloyl esterase (225 mU/mL) and α-l-arabinofuranosidase (211 mU/mL) activities on sugar beet pulp used as carbon source, whereas maximum xylanase (17 U/mL) activity was found during growth on oat spelt xylan [62]. The authors also described higher activities with organic nitrogen sources which yeast extract seem to be the preferable source for the production of all the three enzymes. Concerning the optimized pH, the authors reported highest enzyme activity of FAE during growth at an initial pH of 4, and optimal initial pH of 7 and 6 for highest activity of the enzymes xylanase and α-l-arabinofuranosidase, respectively. The temperature was important for all 3 enzymes to be in the range of 24–30 °C. Crude enzymatic extracts of *P. brasilianum* were also tested for their ability to release free hydroxycinnamic acids and pentoses from seven agricultural substrates (corn cob, corn stover, wheat straw, wheat bran, maize bran, destarched wheat bran, brewer spent grain) and one commercial wheat arabinoxylan. The enzyme mixtures produced by *P. brasilianum* grown on a mixture of brewer’s spent grain (BSG) and sugar beet pulp (SBP), were successfully used to release high value compounds, such as ferulic and *p*-coumaric acids, as well as xylose and arabinose from the tested agro-industrial waste materials indicating the great potential of this strain [62].

Zeni et al. [17] described the optimization and partial characterization of a pectin liase from *P. brasilianum* isolated from tea. Based on factorial experiments and Plackett-Burman design, the authors were able to verify the best conditions for highest pectin lyase (PMGL) activity. The maximum activity reported for PMGL was 9.0 U/mL and the optimal conditions: 33.0 g/L of citrus pectin in the medium composition, 30.0 g/L of yeast extract and 2.0 g/L of potassium phosphate, 180 rpm, 30 °C, 72 h and pH initial 5.5 [17]. The evaluation of the stability of the crude enzyme extract of PMGL was performed by incubating at different pH and temperatures. The results showed optimal conditions of 5.5 and 37 °C, and highest activity of 11.97 U/mL. Later, Zeni et al. [63] also described the partial characterization of polygalacturonase extracts produced by *P. brasilianum*. The maximum polygalacturonase activity detected by the authors was 45.68 U/mL at pH of 5.5 and 37 °C. The partial characterization of the crude enzymatic extract indicated optimum activity at pH 5.5 and 37 °C and temperature stability at 55 °C and pH 4.0 and 5.0. 

Recently, Zeni et al. [64] studied the production and characterization of *P. brasilianum* pectinases aiming to test their potential for industrial application (i.e., fruit juice). The authors performed experimental design and were able to optimize the cultivation conditions (180 rpm, an aeration rate of 1.5 vvm, 30 °C, pH_initial_ of 5.5, 32 g/L pectin, 10 g/L of yeast extract and 0.5 g/L magnesium sulfate and bioproduction for 36 h) in order to achieve highest pectinolytic activity of exo-polygalacturonase (Exo-PG) (53.8 U/mL). The authors also detected pectin methylesterase (6.0 U/mL) and pectin lyase (6.61 U/mL) activities. The results indicated great potential of *P. brasilianum* to be applied in the clarification of juices [64].

Kristian et al. [65] reported the characterization and kinetic analysis of a thermostable GH3 β-glucosidase (BGL) from *Penicillium brasilianum*. In the study, the authors described for the first time a sequence of a BGL from the genus *Penicillium*, and *bgl1* showed highest identity to two BGLs of GH3 family. Both native and heterologous GH3 BGL proteins were obtained from *P. brasilianum*. The BGL showed good stability at elevated temperatures in comparison to Novozym 188, a commercial product with BGL activity and to other fungal GH3 BGLs reported in the literature [65]. The Michaelis-Menten (MM) constants *K*_M_ and *V*_max_ were also determined using both *para*-nitrophenyl-β-d-glucopyranoside (*p*NP-Glc) and cellobiose as substrates and indicated lower affinity for cellobiose compared with the artificial substrate *p*NP-Glc.

Bojarová et al. [66] during the study about the versatility of glycosyl azides as donors in transglycosylation reactions with a representative sample of various glycosidases (α- and β-galactosidases, β-glucosidases, and α-mannosidases) briefly mentioned the production of α-galactosidases obtained from different fungal sources, including *P. brasilianum* CCF 2155. Three enzyme classes, β-galactosidases, β-glucosidases, and α-mannosidases, proved to be good catalysts for synthesis with glycosyl azides [66].

Fill et al. [11] provided insights into the biosynthesis of phenylpropanoid amides (brasiliamides) by *Penicillium brasilianum* found in root bark of *Melia azedarach*. The biosynthetic studies on brasiliamides seem to classify these compounds as phenylpropanoids, which are very uncommon in fungi [67]. The first step in the phenylpropanoid pathway begins with the action of the enzyme phenylalanine ammonia lyase (PAL) to produce cinnamic acid and cinnamic acid-derived compounds, and is frequently associated with plant defense mechanisms against invading microorganisms. The enzymatic experiments were performed in order to verify the PAL activity in the crude extracts of *P. brasilianum* [67]. The authors clearly demonstrated that brasiliamides are biosynthesized by *P. brasilianum* using two l-phenylalanine (Phe) units. In addition, cinnamic acid is produced when Phe is incubated with an enzymatic extract obtained from this fungus, indicating that PAL is active in this extract and may be involved in brasiliamides biosynthesis [67]. Moreover, PAL is one of the few nonhydrolytic enzymes that have important commercial applications [68], being useful for the production of l-phenylalanine (l-Phe) from trans-cinnamic acid through the reverse physiological reaction [69]. In addition, PAL is effective in the treatment of certain mouse tumors [70] and useful in quantitative analysis of serum L-Phe in monitoring patients with phenylketonuria [71] and may be a very interesting target for biotechnological applications.

Another important biotechnological application from *P. brasilianum* was studied by Santos-Fo et al. [72]. The authors investigated the potential production of lipid biodiesel precursors from liquid culture produced by *P. brasilianum* and other endophytic fungi. The extracts from *P. brasilianum* were subjected to acid catalyzed transesterification reactions with methanol; producing the following methyl esters: palmitic acid (26.4%), stearic acid (6.04%), oleic acid (13.9%), linoleic acid (44.6%) and linolenic acid (0.94%), which are considered the most important methyl esters in biodiesel from plants like soybeans [73]. Although the ability to produce the concentration of biodiesel of 50.8 %, *P. brasilianum* did not exhibit methyl ester concentrations of satisfactory levels to be considered as suitable biofuels, especially when compared to soybean biodiesel (90.7%), one of the best known biodiesels made from vegetable sources [74]. 

In recent years, there has been intense interest to investigate the use of nanoparticles for biological applications. Kubo and co-workers [75] reported the formation of microtubules through self-assembly of gold nanoparticles on living fungal biotemplates. They studied the adsorption rates to form microtubules and observed different time for each fungi (*Penicillium brasilianum, Aspergillus aculeatus,* and *Xylaria* sp.), concluding that secondary metabolites and growth media in the fungi metabolism have affected the colloidal gold nanoparticles formation. The time required to form microtubules presented shortest time (5 days) achieved by *Penicillium brasilianum* and longest time (15 days) using *Aspergillus aculeatus*. The authors suggested that the differences in adsorption kinetics are related to secondary metabolites excreted by filamentous fungi, confirming the role played by these metabolites on the deposition process.

The potential of bioremediation of metallic ions as cadmium, cobalt, copper, lithium, lead, and nickel (present in aqueous residues) using both growing and resting cells of *Penicillium brasilianum* and 7 other *Penicillium* species was investigated [74]. Experiments were carried out in three binary mixtures (copper and nickel, cadmium and lithium, cobalt and lead), as well as lead in addition to cadmium, copper, nickel, and lithium constituting other mixtures. *P. brasilianum* exhibited tolerance toward the following metallic ions: nickel, lithium, cobalt, and lead presenting removal levels superior to the concentration of 500 μg/mL. Among evaluation of *P. brasilianum*’*s* activity over heavy metals and its absorption capacity, the most significant feature was the bioremediation of 74.4% of lead ion removed when this metal was present in a binary mixture containing lithium. For this reason, the authors considered *Penicillium brasilianum* as a promising alternative for the development of new technologies for the treatment of water containing residues of metals. *Penicillium brasilianum* has also been extensively studied concerning the biotransformation of non-natural substrates due to its great enzymatic potential. Biotransformation can be defined as the use of biological systems to produce chemical changes on compounds that are not their natural substrates [76]. A substrate can be modified by transforming functional groups, with or without degradation of carbon skeleton. Such modifications may result in the formation of new and useful products which may not be easily prepared by chemical methods [77]. Hence, microbial biotransformation process has become a novel alternative method to obtain high-value bioactive products. Researchers have described *P. brasilianum*’*s* ability to modify non-natural substrates chemical structures with a high degree of stereospecificity as will be described in the next paragraphs.

Fill et al. [78] investigated the oxidative potential of the fungus *P. brasilianum* using 1-indanone as a substrate to track the production of monooxygenases. The authors report that *P. brasilianum* was able to convert the substrate into dihydrocoumarin with the classical Baeyer-Villiger reaction regiochemistry, and (−)-(*R*)-3-hydroxy-1-indanone with 78% ee, Byproducts of different biotransformation reactions were also detected in the experiments, especially compounds resulting from lipase and SAM activities, indicating that 1-indanone is a good probe molecule to track different enzymes in fungi [78], exhibiting *P. brasilianum* as a potential microorganism for biotransformation reactions.

The microbial diversification of racemic Diels–Alder endo-cycloadducts by whole cells of *Penicillium brasilianum* was recently demonstrated by Din and co-workers [79]. The authors described the use of *P. brasilianum* as a catalyst in order to induce the biotransformation of Diels-Alder endo-cycloadducts obtained from the reaction of the dienes, cyclopentadiene and 2,3-dimethylbutadiene with *para*-benzoquinones. The experiments resulted in 15 biotransformed products, being eight of them novel terpene analogs. The fungus was able to perform oxidation and ring closure reactions, reduction of the C=C or C=O in α,β-unsaturated system, and allylic hydroxylations [79].

In 2015, Horn and researchers published the genome sequence of the fungus *Penicillium brasilianum* MG11 (soil isolate). The final assembly resulted in a genome comprising 87 scaffolds and the annotation predicted 11,432 genes and 12,343 transcripts [80]. Functional annotation suggested names for 4758 transcripts and assigned 438 transcripts to 36 putative secondary metabolite gene clusters. Fill et al. [47] also described the great potential for secondary metabolite production coded in the genome of *P. brasilianum* LaBioMMi 136. The authors described that this strain had its genome sequenced, although the data is not yet published. The related information submitted to AntiSmash analysis indicate that *P. brasilianum* has 42 putative biosynthetic gene clusters containing, among others, 22 backbone genes, of which 12 are nonribosomal peptide synthetases (NRPSs) [47].

## 5. Conclusions

Different strains of *Penicillium brasilianum* have been isolated worldwide from a variety of environmental sources, from soil isolates, to endophytes and phytopathogenic. Based on the chemical studies reviewed here, *P. brasilianum* is considered a remarkable microorganism with great potential for secondary metabolite production with a variety of carbon skeletons, and most of them exhibiting interesting biological activities. Meroterpenenes and brasiliamides are the main chemical constituents, however, polyketides, diketopiperazines, alkaloids and cyclodepsipeptides with potential biological activities have also been described. For decades, mainly analytical and chemical methods gave access to secondary metabolites, nowadays genomics-based methods offer complementary approaches to find, identify and characterize such molecules. Furthermore, functional genome analysis seems to indicate an even greater potential for secondary metabolite discovery in this species leaving an open door for further chemical and biosynthetic studies. 

*Penicillium* is one of the most used genera in biotechnology and, *Penicillium brasilianum* is also a very important biotechnological target for interesting enzyme production, bioremediation and biotransformation processes. Important hydrolytic enzymes were described by this species indicating the great potential of this fungus to be applied in industries i.e. clarification of juices. Due to this great potential, *P. brasilianum* continue to be an interesting target for metabolic and enzymatic studies and remains underexplored.

## Figures and Tables

**Table 1:** Secondary metabolites produced by *Penicillium brasilianum*.

Compound	Chemical Structure	Molecular Formula	Bioactivity	Reference
Austin **1**	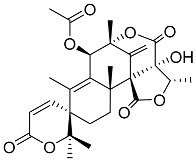	C_27_H_32_O_9_	Antibacterial, Antagonists on neuronal nicotinic acetylcholine receptors	[13,21,24,25]
Austinol **2**	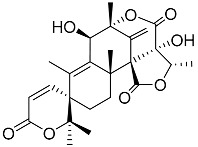	C_25_H_30_O_7_		[24]
Dehydroaustinol **3**	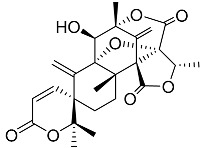	C_25_H_28_O_8_		[24]
Austinolide **4**	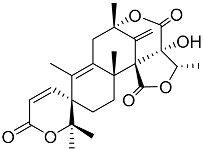	C_22_H_26_O_9_	Antibacterial	[9,11,13,21,26,27]
Austinoneol **5**	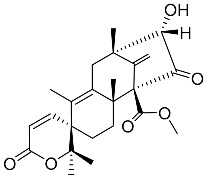	C_24_H_30_O_6_	Antibacterial	[27]
Dehydroaustin **6**	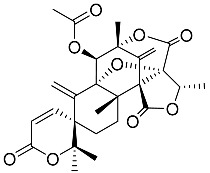	C_27_H_30_O_9_	Antagonists on neuronal nicotinic acetylcholine receptors, Insecticide	[13,24,25,28]
Acetoxydehydroaustin **7**	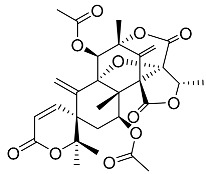	C_29_H_32_O_11_	Antibacterial, Antagonists on neuronal nicotinic acetylcholine receptors, Insecticide	[13,24,25,27,29]
Neoaustin **8**	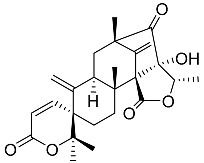	C_25_H_30_O_6_	Antibacterial	[24,27]
Isoaustinone **9**	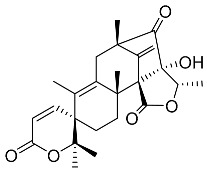	C_25_H_30_O_6_	Antibacterial	[9,21,26,27]
Preaustinoid A **10**	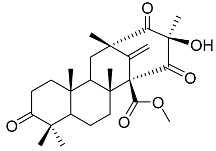	C_26_H_36_O_6_	Antibacterial, Inhibition of Caspase-1	[9,30]
Preaustinoid A1 **11**	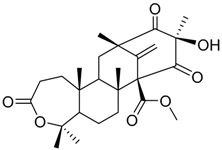	C_26_H_36_O_7_	Inhibition of Caspase-1	[9,13,21,26,27,30]
Preaustinoid A2 **12**	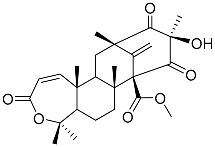	C_26_H_34_O_7_		[26]
Preaustinoid A3 **13**	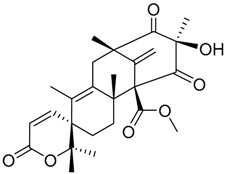	C_26_H_32_O_7_		[21]
Preaustinoid B **14**	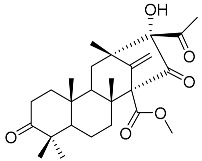	C_26_H_36_O_6_	Antibacterial	[9]
Preaustinoid B1 **15**	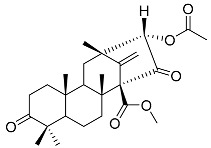	C_26_H_36_O_6_		[26]
Preaustinoid B2 **16**	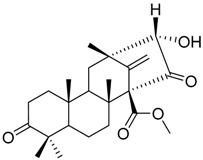	C_24_H_34_O_5_		[9,11,13,21,26,27]
Brasiliamide A **17**	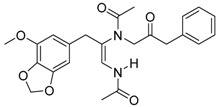	C_24_H_26_N_2_O_6_	Bacteriostatic, Convulsive activity	[11,12,20]
Brasiliamide B **18**	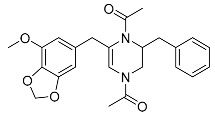	C_24_H_26_N_2_O_5_	Antibacterial, Convulsive activity	[11,12,20]
Brasiliamide C **19**	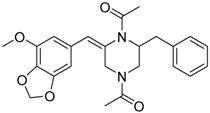	C_24_H_26_N_2_O_5_	Convulsive activity	[11,12,20]
Brasiliamide D **20**	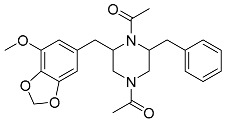	C_24_H_28_N_2_O_5_	Convulsive activity	[11,12,20]
Brasiliamide E **21**	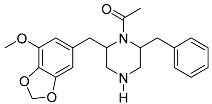	C_22_H_26_N_2_O_4_		[11,12,20]
Brasiliamide F **22**	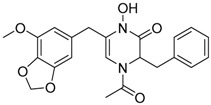	C_22_H_22_N_2_O_5_	Antibacterial	[11,20]
Penicillic acid **23**	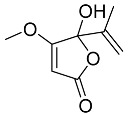	C_8_H_10_O_4_	Antibacterial, herbicide, Inhibit germination of fungal spores	[13,31]
d-mannitol **24**	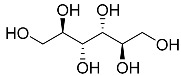	C_6_H_14_O_6_	Antibacterial Anti-hypertensive	[13,32]
Verruculogen **25**	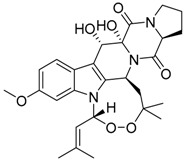	C_27_H_33_N_3_O_7_	Tremorgenic, Antibacterial, Week antiparasitary	[9,23,24,33,34]
Verruculogen TR-2 **26**	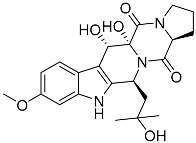	C_22_H_27_N_3_O_6_		[22]
Verruculogen TR-2 epimer **27**	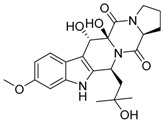	C_22_H_27_N_3_O_6_		[22]
Isoroquefortine C **28**	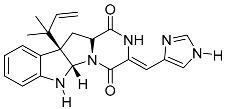	C_22_H_23_N_5_O_2_	Antifungal	[35]
Griseofulvin **29**	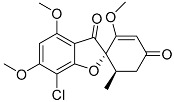	C_17_H_17_ClO_6_	Antibacterial, Antifungal	[35]
Ergosterol peroxide **30**	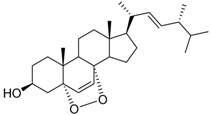	C_28_H_44_O_3_		[35]
3β-Hydroxy-(22*E*,24*R*)-ergosta-5,8,22-trien-7-one **31**	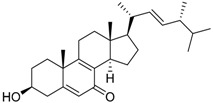	C_28_H_43_O_2_		[35]
Cerevisterol **32**	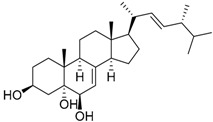	C_28_H_46_O_3_		[35]
(22*E*,24*R*)-6β-Methoxyergosta-7,22-diene-3β,5α-diol **33**	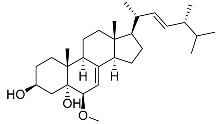	C_29_H_48_O_3_		[35]
JBIR 113 **34**	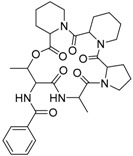	C_31_H_42_N_5_O_7_		[36]
JBIR 114 **35**	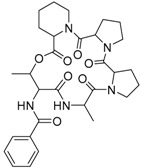	C_30_H_39_N_5_O_7_		[36]
JBIR 115 **36**	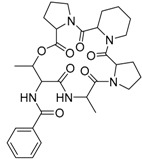	C_30_H_39_N_5_O_7_		[36]
Spirohexaline **37**	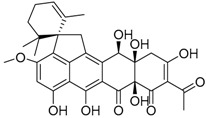	C_31_H_32_O_10_	Antibacterial,	[23,24]
Viridicatumtoxin **38**	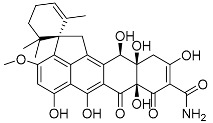	C_30_H_31_NO_10_	Antibacterial, Antifungal, Cytotoxic against lymphocytic leukemia	[23,24,37]
Paraherquonin **39**	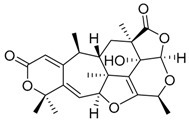	C_24_H_28_O_7_	-	[38]
Berkeleydione **40**	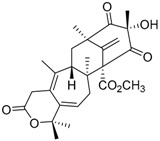	C_26_H_32_O_7_	Inhibition of Metalloproteinase- 3 and Caspase-1	[38]
